# The activities of hospital nursing unit managers and quality of patient care in South African hospitals: a paradox?

**DOI:** 10.3402/gha.v8.26243

**Published:** 2015-05-11

**Authors:** Susan J. Armstrong, Laetitia C. Rispel, Loveday Penn-Kekana

**Affiliations:** 1 Centre for Health Policy & Medical Research Council Health Policy Research Group, School of Public Health, Faculty of Health Sciences, University of the Witwatersrand, Johannesburg, South Africa; 2 Department of Nursing Education, School of Therapeutic Sciences, Faculty of Health Sciences, University of the Witwatersrand, Johannesburg, South Africa; 3 Department of Infectious Disease Epidemiology, London School of Hygiene and Tropical Medicine, London, United Kingdom

**Keywords:** nursing unit managers, quality of care, hospitals, time and motion, South Africa

## Abstract

**Background:**

Improving the quality of health care is central to the proposed health care reforms in South Africa. Nursing unit managers play a key role in coordinating patient care activities and in ensuring quality care in hospitals.

**Objective:**

This paper examines whether the activities of nursing unit managers facilitate the provision of quality patient care in South African hospitals.

**Methods:**

During 2011, a cross-sectional, descriptive study was conducted in nine randomly selected hospitals (six public, three private) in two South African provinces. In each hospital, one of each of the medical, surgical, paediatric, and maternity units was selected (*n*=36). Following informed consent, each unit manager was observed for a period of 2 hours on the survey day and the activities recorded on a minute-by-minute basis. The activities were entered into Microsoft Excel, coded into categories, and analysed according to the time spent on activities in each category. The observation data were complemented by semi-structured interviews with the unit managers who were asked to recall their activities on the day preceding the interview. The interviews were analysed using thematic content analysis.

**Results:**

The study found that nursing unit managers spent 25.8% of their time on direct patient care, 16% on hospital administration, 14% on patient administration, 3.6% on education, 13.4% on support and communication, 3.9% on managing stock and equipment, 11.5% on staff management, and 11.8% on miscellaneous activities. There were also numerous interruptions and distractions. The semi-structured interviews revealed concordance between unit managers’ recall of the time spent on patient care, but a marked inflation of their perceived time spent on hospital administration.

**Conclusion:**

The creation of an enabling practice environment, supportive executive management, and continuing professional development are needed to enable nursing managers to lead the provision of consistent and high-quality patient care.

Improving the quality of health care delivery is an important global priority (1). Around the world, the purpose of health care quality improvement initiatives is to ensure patient safety, improve clinical effectiveness, and promote public accountability (2). In South Africa, the government has established an independent quality of care regulator, the Office of Health Standards Compliance (OHSC), as part of an overall suite of health sector reforms that aim to achieve universal health coverage (3). The aims of the OHSC are to protect and promote the health and safety of health service users, through the effective management of patient complaints and the enforcement of compliance to prescribed norms and standards (3).

There is well-documented evidence globally that the number, competencies, and effectiveness of nurses are critical in determining the quality of care in hospitals and the nature of patient outcomes (4–14). Nursing unit managers play a key role in coordinating patient care activities and in ensuring safety and quality care in hospital wards. These unit managers are professional nurses registered with the South African Nursing Council (SANC), with at least 4 years of nursing training, and extensive clinical experience. The nursing unit manager (sometimes referred to as ‘charge nurse’ or ‘operational manager’) is responsible for the management of: nursing care to patients; all nursing staff within the unit; and the resources associated with health care delivery in the unit (15). These unit managers in both the private and public health sectors are held accountable for the quality of patient care in their units or wards, and they enter into performance management agreements that outline their operational management responsibilities.

Notwithstanding the importance and commonality of time as an economic resource available to all managers (16), most of the time and motion studies have been conducted among doctors and nurses providing direct care to patients (17–26). The focus of these studies has been on: the relationship between nursing time and quality of care (20, 23, 27); interruptions in nursing tasks and the association with adverse clinical events (21, 28); or to determine the time taken for various nursing tasks in specific nursing units to enable staff planning (17–19, 23–26, 29). The findings of these studies suggest that there is a positive association between the amount of time nurses spend with patients and patient or nurse satisfaction, as well as patient outcomes and safety (17–21). Despite the voluminous literature on time and motion studies (22), the majority of these studies are in high-income countries. Furthermore, with the exception of a 1934 study that focused on ‘head nurses’ in the Unites States (30), we could not find recent studies that focus specifically on the nursing unit manager or studies that concentrate on a low- and middle-income country (LMIC) setting. In light of this dearth of empirical evidence and the major emphasis of the South African government on quality of care, this paper examines whether the activities of nursing unit managers facilitate the provision of quality patient care in selected South African hospitals. The specific study objectives were to: record the time taken by nursing unit managers on various activities in the hospital ward; determine the perceptions of these unit managers regarding their own time management; and examine whether the range and diversity of nursing unit manager activities facilitate quality of care in their units. The findings reported in this paper are part of a larger research programme to examine the relationship between nursing management and quality of care.

## Methods

### Ethical considerations

The University of the Witwatersrand Human Research Ethics Committee (Medical) granted ethical approval for the study. The public and private health care authorities in the two study provinces also provided study approval. All participants received a study information sheet and were required to sign an informed consent form to indicate their willingness to participate in the study.

### Research setting

Two South African provinces, Gauteng and the Free State, were selected purposively because of geographical proximity to the researchers, health authority approval, and budgetary constraints. Gauteng is the most urbanised and densely populated province with a population of 12.2 million, while the Free State province is a mixed urban–rural, largely agricultural province with a population of 2.7 million (31).

### Sampling

The study sample was not intended to be representative of all unit managers in the country, but rather to explore issues of nursing management, quality of care, and clinical governance at hospital unit level that could serve as a basis for future research. Budgetary constraints also influenced the number of hospitals and units selected for in-depth study.

In the Gauteng province, three hospitals were selected from the public health sector, and three from the private health sector. In the case of the public sector hospitals, one hospital was selected randomly from each cluster of tertiary, regional, and district hospitals (total of three hospitals). All specialised psychiatric and tuberculosis hospitals were excluded. In the case of the private hospitals, only non-specialised hospitals with more than 100 beds were included in the sampling frame. One hospital was selected randomly from each of the three major private hospital groups in South Africa (total of three hospitals).

In the Free State province, only public hospitals were included, with one hospital selected from each of the cluster of tertiary, regional, and district hospitals (total of three hospitals). At each hospital, one medical, surgical, paediatric, and maternity (labour and post-natal) unit was selected (total four or five units per hospital depending on the size of the hospital). In hospitals with more than one of these units, a simple random sampling technique was used to select the unit. A total of 36 units were selected.

The final sample consisted of six public sector hospitals out of a total of 54 public hospitals, and three private hospitals out of a total of 99 private hospitals in the two study provinces.

### Study participants

All the nursing unit managers (*n*=36) in charge of the selected hospital units were invited to participate in the study.

### Data collection and analysis

The data was collected over a 4-month period from September until December 2011. There were two components to the data collection: a time and motion study which involved continuous and independent observation of unit managers’ work activities; and semi-structured in-depth interviews to determine the managers’ perceptions of their own time use and management.

Following informed consent, each selected unit manager was observed by a trained, nurse-field worker for a period of 2 hours on the survey day (1 hour in the morning and one in the afternoon) and the activities recorded on a minute-by-minute basis. The fieldworkers were trained to be as discreet as possible and not to interfere with the work of the unit manager.

Hence, there was a total of 72 hours of observation. The activities were entered into Microsoft Excel and coded into categories. The research team used thematic content analysis to develop the themes or categories (32). One researcher read through all the recorded activities of nursing unit managers to get an overview of the findings and to highlight important issues that emerged (32). A distinction was then made between the main, unique, and other themes that emerged from the data. A second researcher read a sample of the recordings independently and also recorded the issues that emerged from the data. The researchers met to discuss the emerging themes in light of the study objectives and to reach agreement on these themes or categories of activities (Table 1).

**Table 1:** Categories of nursing managers’ activities identified from time and motion study

Category	Brief description
Patient care	Includes the assessment of patients or providing, checking, directing, discussing, organising, or coordinating care
Hospital administration	Management or quality assurance meetings, discussion with individuals, private or corporate organisations
Patient administration	Admissions and discharges, completing or checking nursing records, checking medical or essential equipment
Education	Includes patient or junior staff education, or organising continuing professional development activities
Communication and support	Includes telephone calls and communication or support provided to all categories of staff (doctors, nurses, other staff members), patients, their relatives or visitors, students
Equipment and stock management	Ordering, checking, receiving, distributing, or locating items or consumables
Staff management	Directing, correcting, orientating, sourcing, allocating and delegating, conflict resolution, and staff evaluation
Miscellaneous (other)	Walking around and seeking items, ward hygiene, tidying, maintenance and support services, breaks – lunch/tea/rest

After agreement was reached on the categories, the activities were coded and analysed according to the time spent on activities in each category. Microsoft Excel was used to analyse the data and to determine the time spent on the various categories of activity by each unit manager and the average time spent on each category of activities when the data was combined for all nursing unit managers.

A semi-structured in-depth interview was conducted with each nursing unit manager, who was asked to think about and reflect upon the previous working day. The manager was asked to indicate which three activities or tasks took up most of their time, and whether there were any activities or tasks that they wanted to get done, but just could not find the time to do. They were also asked whether there were daily tasks that take up too much of their time; and/or tasks that they do not have time to get round to. Each interview was recorded and transcribed verbatim. The information related to their perceptions of the activities in which they had engaged the previous day were analysed using thematic content analysis (32). The activities mentioned by the nursing managers were listed, as well as their estimates of the time spent on each activity. The self-reported information obtained from the inductive analysis was compared with the codes derived from the time and motion study described above, and found to be similar. The research team listed additional emerging themes separately.

Following the generation of themes, a workshop was held with the nursing unit managers. They were asked to comment on the analysis of their daily activities, the categories and themes, and to reflect on whether the results obtained represented their management experiences. The feedback meeting provided space for reflectivity and ensured the credibility of the research findings.

## Results

### Time and motion study

**Fig. 1:**
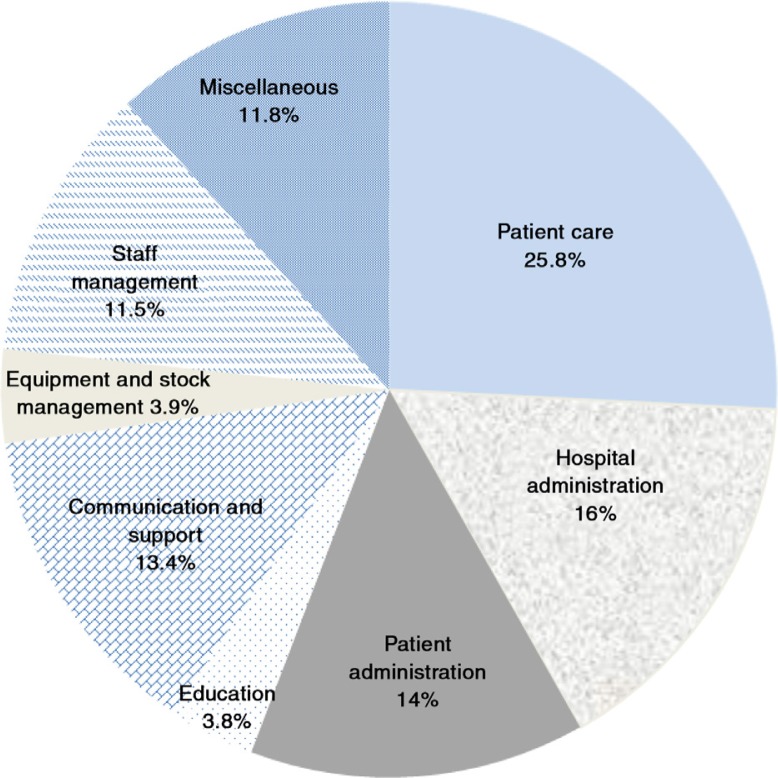
Nursing unit managers’ activities in the time–motion study.

All nursing unit managers agreed to participate in the study. Eight categories of activity were identified from the observations of the unit managers’ activities. The time and motion study found that nursing unit managers spent 25.8% of their time on direct patient care, 16% on hospital administration, 14% on patient administration, 3.6% on education, 13.4% on support and communication, 3.9% on managing stock and equipment, 11.5% on staff management, and 11.8% on miscellaneous activities (Fig. 1).

As can be seen from Fig. 1, unit managers spent 25.8% of their time on direct patient care (Fig. 1), which was more time spent than on any other single group of activities. These activities took place at the patient's bedside and included direct patient care such as positioning the patient, assisting new mothers to breastfeed, administering analgesia, or assisting patients to eat. The nursing manager indicated that they felt obliged to provide this basic nursing care, rather than delegate it to a more junior nurse. The provision of direct patient care typically occurred while on the way to perform a more advanced nursing task such as administering intravenous medication or monitoring a blood transfusion. Much of the time spent coordinating care resulted from the doctors’ ward rounds, with the manager ensuring that the treatment was given or that X-rays and blood tests were done. In the public hospitals where a doctor did a ward round once a day, the unit managers were able to organise all treatment at one time, which greatly assisted with time management. This task became very time consuming in the private hospital units where private doctors may have one or two patients in the ward and arrive at a time that suited them which resulted in the unit managers having to stop other activities to deal with the instructions of a single doctor.

Nursing unit managers spent an average of 16% on hospital administration. Meetings took up a significant part of the unit manager's time, particularly in the public hospitals where all the unit managers would be called to meetings with the nursing service manager, lasting more than 1 hour at a time. At the time of the fieldwork, the one province was expecting accreditation visits to be carried out within the month. The time spent on quality assurance activities at these provincial hospitals was significant with one unit manager spending her entire day making sure that all hospital records were ready for the inspection.

Only 3.6% of unit manager's time was spent teaching staff and students despite all of the selected hospitals being training facilities. Nursing students were present in many of the wards but the unit managers did not appear to see teaching as part of their responsibilities. However, the managers corrected staff and students when they observed the provision of inadequate care.

Unit managers spent an average of 3.9% on managing stock and equipment, but this varied depending on the presence of a ward clerk in the unit. One hospital had devolved the management of stock and equipment to the responsible department and did not hold the unit manager accountable for managing stock. For example, in the case of medicines, the pharmacist was responsible for checking stock levels, ordering, and supplying the appropriate medicines.

The study found that 11.8% of the nursing unit manager's time was spent on miscellaneous activities, with almost half of this proportion (48%) spent on walking and in search of stock, keys or other staff members. In several of the units, the unit manager was the only person entrusted with the stock and medicine keys resulting in her having to walk back to the cupboards every time a junior staff member needed medication or other items for patient treatment. Unit managers preferred to record information in patient files in their offices or at the nurses’ stations, hence time was spent time fetching these patient files. In the public hospitals, unit managers collected medicines or consumables personally from the pharmacy or store-rooms respectively, because they reportedly had greater powers of persuasion, compared to junior staff members who were less likely to obtain these necessary items.

### Perception of nursing managers of time spent on different activities

**Fig. 2:**
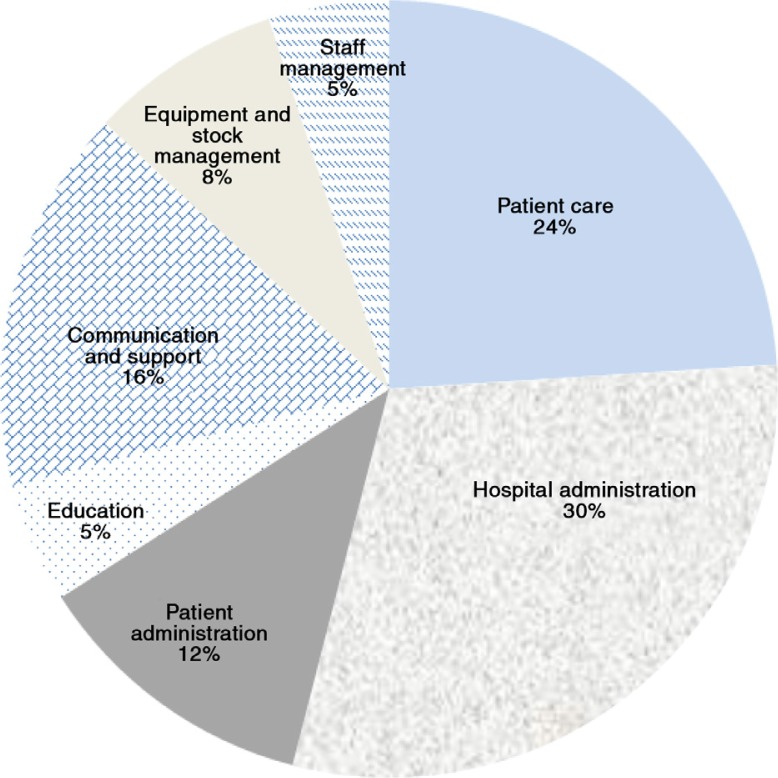
Nursing unit managers’ perceptions of time spent on different activities.

Figure 2 shows the perceptions of nursing managers of the time spent on different activities.

As can be seen from Fig. 2, 24% of the activities identified by nursing unit managers related to patient care, 30% to hospital administration, 16% support and communication, 12% on patient administration, 8% stock and equipment, with the remaining time spent on staff management, and education.

### Interruption experienced by nursing managers

The time and motion study found that a nursing unit manager had to deal with 36 different types of activities in 1 hour. In one instance, it took a nursing manager 30 minutes to make an entry in a patient record, because of all the interruptions that ranged from answering the telephone, responding to patient visitors, providing support to doctors or assisting junior nurses. In order to prevent these interruptions, unit managers frequently completed their management reports in an area away from the unit.

### Factors influencing unit managers time utilisation and management

The interviews revealed that several issues influenced the time spent by nursing managers on the various activities, as well as the management of their time. These were: health workforce issues (shortages, staff performance); sub-optimal communication; resource constraints; and unplanned activities and interruptions. Each of these is explored briefly.

A recurring theme that emerged from the interviews was issues related to the health workforce, particularly staff shortages and sub-optimal staff performance, the latter influenced by their perceived competencies. Unit managers particularly in the public hospitals made several references to staff shortages which reportedly interfered with their management responsibilities, illustrated by the following comments.

I was so short staffed I didn't do any managerial duties. (Respondent 15, public paediatric unit)

I didn't get round to off duties. I was particularly understaffed yesterday as the staff nurse was on a [training] course and students were in class. (Respondent 2, public surgical unit)

The performance of staff was another concern for unit managers. Absenteeism of nurses with little or no notice and seemingly suspect reasons caused a lot of frustration among the nursing unit managers. A private hospital maternity unit manager gave an example of the incompetence of a nurse in her unit, whom she felt she could not trust, and this impacted on the time she (the nursing manager) spent on various activities.

I didn't have time to sit and talk with a nurse who other staff members have been complaining that she isn't doing her work. I need to deal seriously with this issue. Keep a record of complaints etc. Need to build a case – and work out what to do. Letting it linger could lead to a problem with a patient. (Respondent 28, private maternity unit)

Nursing managers in both public and private hospitals reported problems of sub-optimal communication, particularly poor record-keeping that influences patient care, as well as inadequate communication between doctors, patients, and nurses. A manager from a private paediatric unit said:

We (have to) compensate for the fact that the doctors can't explain clearly to the patients. (Respondent 35, private paediatric unit)

This same problem was reported in another private hospital paediatric unit where the manager said:

Supervision- you waste so much time … checking records that keep being wrong. (Respondent 15, private paediatric unit)

Although the nature and magnitude of resource constraints were different between public and private hospitals, all the unit managers complained that this aspect made their jobs more challenging. In both the public and private hospitals, unit managers complained about the time taken, and difficulties, of finding beds. The bed shortages resulted at times in premature discharge of some patients, and admission of new, very ill patients.

I spent 2 hours with the CEO [chief executive officer] trying to sort out beds and equipment issues. (Respondent 4, public post-natal unit)

In the private sector, unit managers complained about the time spent in finding agency nurses.

Finding beds and finding agency staff takes a lot of time. (Respondent 31, private paediatric unit)

I didn't have time (for important issues because I was) checking agency staff – hours worked, salaries paid etc. (Respondent 28, private post-natal unit)

### Perceptions of unplanned activities and interruptions

All unit managers raised unplanned activities and interruptions as a major problem. Some of these were unforeseen, such as the suicide of a staff member or medical emergencies such as cardiac arrests, unplanned caesarean sections or transfer of patients to intensive care units. These were common to both public and private hospital unit managers, as can be seen from the comments below:

I spent lots of time with a patient who needed to be sent to ICU because she didn't have an [urine] output. (Respondent 16, public post-natal unit)

I had a resuscitation that I didn't expect – it took 2 hours of my time. (Respondent 29, private medical unit)

I had two critically ill babies and I struggled to get a doctor. (Respondent 15, public paediatric unit)

Medical emergencies had occurred in four of the units on the day prior to the fieldwork.

Unit managers also complained about the time taken for orientation of new staff members or community service nurses, record audits, and quality assurance activities. They also indicated that telephone enquiries from relatives or staff, in the absence of support staff such as clerks, take up a great deal of time and detract from patient care and management activities.

More than one third of nursing unit managers complained about administration and that meetings take up too much of their time. One said that there is so much administration that she feels like a clerk at time, while others said the following:

There is so much admin, and meetings. For the ward and hospital, I spend a lot of time travelling between hospitals and capturing data for the DHIS [district health information system]. We could do with some secretaries and supervisors for support staff. (Respondent 23, public paediatric unit)

We keep talking about the problem – but it is never solved - the meetings, meetings, meetings, take up a lot of time. (Respondent 12, public maternity unit)

The executive nurse manager of one of the hospitals was also in charge of another hospital, 50 km away. This meant that every time a meeting of the unit managers was called, at least one set of nursing managers had to travel, resulting in an entire day away from their units.

## Discussion

The time and motion study found that on average, nursing unit managers in the selected hospitals spent around one quarter (25.8%) of their time on patient care activities. This was similar to the proportion (26%) that the unit managers estimated to spend on patient care. When probed about the amount of time spent on patient care, they indicated that they had to care for the patients themselves as there was no alternative, either because of the complexity of the nursing task or because of staff shortages. We could not find similar studies focusing on nursing unit managers in other LMIC settings. Although not directly comparable, a 2008 study in a medical ward in Australia found that nurses spent 33.2% on direct patient care (33), while another Australian study in two wards in a teaching hospital found that nurses spent 37% of their time with patients (20). Similarly a Belgian study found that nurses spent 32.2% of their time on direct patient care (24), while a similar proportion of 32.8% was found in a Montreal hospital study among surgical nurses (17). The 1934 US study found that ‘head nurses’ or unit managers spent around 15.6% of their time on direct patient care (30), while another US study found that nurses spent 44% of their time on patient care (19).

There is no norm of the proportion of time that nursing unit managers should spend on patient care. Although it is encouraging that unit managers spent such a large proportion of their time on patient care, it could mean that they may not have enough time to carry out their primary management responsibilities. These duties include the strategic management of patient care activities in the unit (e.g. co-ordination of patient care, overseeing quality of care initiatives), teaching and mentoring of students and junior staff, and the management of human resources, finances, equipment, pharmaceuticals, and other resources.

Although unit managers spent 25.5% of their time on patient care, our study found that they engaged in 36 different tasks per hour, averaging less than 2 minutes per task. Although this was better than the average of 72.3 tasks per hour in an Australian study, our study finding implies that unit managers perform numerous, fragmented tasks of short length. This could mean that changes in a patient's condition may not be noticed. Nursing scholars have argued that fragmented patient time is not conducive to nursing surveillance (34, 35), which is considered essential for quality of care, especially the identification and prevention of medical errors and adverse events (35).

In our study, unit managers spent a sizeable proportion of their time on hospital (16%) or patient (14%) administration. The perceptions of nursing managers were that they spent around 30% of their time on hospital administration, with meetings being the most frustrating aspect mentioned. The discrepancy between actual (observed) and perceived proportion of time spent on hospital administration could be due to different perceptions of roles and responsibilities among nursing managers, lack of knowledge on these roles and responsibilities, and/or because they found administration to be less satisfying, than providing direct patient care. Other studies have found that the proportion of time spent on administration ranged from a low of 6% in an Australian study (33) to 33.1% in the 1934 study of ‘head nurses’ (30).

One of the most striking aspects of this study was the number of interruptions the unit managers experienced. An interruption is defined as a ‘a break in performance that occurs in response to a source that is external or internal to a person, e.g. daydreaming, suspension of the initial task, performance of another task, and resumption of the primary task’ (36, p. 2). While the interruptions and the unplanned activities were not unexpected, the nature of some of these gave insight into the practice environment of the unit managers. Other studies have demonstrated that task interruptions affect patient safety as they could increase medication errors or adverse events (21, 27, 28, 36). Interruptions also have a negative impact on work procedures, work flow, ability to concentrate, reflective processes, and interaction with patients (28, 37).

The study found that nursing unit managers spent 11.5% on staff management, but the self-assessment of the unit managers was that they spent only 5% on this aspect. The 1934 US study found that the head nurses spent 22.5% on ‘supervision’ (30), but the studies are not directly comparable. At the same time, the interviews revealed that unit managers seem to shy away from active performance management of staff, rather than deal with problems of absenteeism or complaints about staff performance directly.

We also found that 11.8% of time was spent on miscellaneous activities (Fig. 1), but this did not feature as a category mentioned by the unit managers. As indicated, half of the time is spent simply on walking and looking for stock, keys or other staff members. This suggests a rather centralised system of unit management, rather than a more distributed leadership model.

During the interviews, unit managers discussed the factors impacting on their time management and they spoke passionately about their frustrations with time-consuming activities, which they believe have a negative impact on the quality of patient care in their units. The four major themes were health workforce issues, resource constraints, sub-optimal communication, and interruptions and unplanned activities.

There are limitations of this study, which was undertaken among 36 nursing unit managers in nine hospitals. The study sample is small, and no analysis of variance was possible between the public and private hospitals, urban and rural hospitals, or among different hospital units. The findings may have limited generalisability to other hospitals, both in South Africa or other LMIC settings. The observation period was relatively short, and this may have influenced the results obtained. Although the fieldworkers tried to be discreet during the observation of nursing managers, their presence could have influenced the behaviour of the nursing managers. The data collection instruments were designed for our study aims, making cross-study comparability difficult. The in-depth interviews elicited self-reported information from nursing unit managers on their time management and the time spent on different types of activities. The results may reflect social desirability bias, with nursing unit managers over-estimating the time spent on hospital administration. We did not ask nursing managers about their perceptions of their roles and responsibilities, or what they considered to be the ideal time to spend on different types of activities. We are unable therefore to determine whether the results obtained could be explained by possible differences in unit managers’ perceptions of their roles and responsibilities. This is an area for further research.

Nonetheless, there are several study strengths. This was one of the first studies in South Africa and in a LMIC setting to examine the work activities of nursing unit managers, and to combine direct observation with in-depth interviews with these managers. The study findings could be used as a baseline for larger, national studies on clinical governance at hospital unit level, and the methods could be validated and/or enhanced in future research. The study findings were validated during a workshop with the nursing managers, thus giving voice to these individuals. The interaction with nursing managers also allowed the nursing managers to be reflective, and to learn from the experiences of other managers, both in the public and private health sectors.

The study findings provide a glimpse into the difficulties of achieving a quality of care revolution as envisaged by the establishment of the independent OHSC in South Africa. Nursing unit managers are the custodians of professional nursing practice, quality patient care, and safety in hospitals. Hence, the issues identified in this study must be addressed to ensure that they fulfil their clinical leadership role in hospitals.

In the short-term, support from the hospital executive management, particularly the executive nursing manager, is critical. At the very least, a forum that brings together the different unit managers in each hospital would be a step in the right direction. Such a forum would allow for sharing of good practices, peer-to-peer mentoring, and the development of solutions to common problems such as dealing with difficult staff members or mechanisms to improve communication among all members of the health team.

In those study hospitals that employed ward clerks or that used an electronic patient information system, the workload of unit managers was eased considerably. In the public hospitals, the idea of ward clerks to relieve the unit manager of routine administrative work has been revived, but implementation has been patchy. The employment of ward clerks would be an important intervention. Another approach, which appears valuable in one province and in the private health sector, was the requirement that medicines and supplies were managed by the relevant departments and not by the unit manager. Unit managers were involved in determining minimum and maximum stock levels, after which other categories of staff keep control of ordering and delivery of stock.

In the private sector, the employment of a ‘shift leader’ appears to assist with the problem of interruptions of the nursing unit manager. This individual is a registered nurse who takes responsibility for coordinating patient care, thus allowing the unit manager to concentrate on her management or leadership responsibility. Although this solution depends on the budget availability for an additional registered nurse, the additional cost of employing a shift leader may be off-set by an increase in patient satisfaction and a reduction in adverse events. However, this is an area for further research.

We recommend that continuing professional development programmes be developed for nursing unit managers to enhance their management skills and abilities. These should include, *inter alia*, training or coaching in delegation, conflict management (doctors, fellow nurses, patients, relatives of patients), human resource management, time management, organisational skills, leadership for quality assurance, as well as unit management. The roles and responsibilities associated with unit management should also be emphasised. These aspects should also be addressed in the pre-service education and training of registered nurses.

Although our study concentrated on the nursing unit manager, our study findings suggest that problems in the practice environment affect all categories of staff, including junior nurses. In the primary health care environment, a survey found that positive practice environments influence the job satisfaction of managers (38). Globally, there has been strong advocacy for positive practice environments that enable high quality of care to be delivered (5, 9, 39). In South Africa, there is an enabling policy environment for the delivery of quality of care (3, 40), but much needs to be done to ensure actual implementation of these policies that will create positive practice environments.

## Conclusion

The study examined whether the activities of nursing unit managers facilitate the provision of quality patient care in South African hospitals. The answer is not straightforward. On the positive side, the study found that 25.5% of the time of the nursing unit managers is spent on patient care. Although this proportion compares well with the findings of studies among nurses providing direct care in high-income countries (17, 19, 20, 24, 33), this proportion might not be appropriate in light of the core management responsibilities of nursing unit managers. The study also found that unit managers experienced numerous interruptions, performing many short, fragmented tasks. A significant proportion of time was spent on miscellaneous activities, which provides an opportunity for intervention. The practice environment with staff shortages and performance problems, resource constraints, sub-optimal communication, and unplanned activities exacerbate the difficulties of the unit manager to provide leadership for the delivery of high-quality patient care.

This study has highlighted the work activities of nursing unit managers, and has explored its relationship to the provision of quality care in selected hospitals. The creation of an enabling practice environment, supportive executive management, and continuing professional development are needed to enable nursing managers to lead and oversee the provision of consistent and high-quality patient care in these South African hospitals.
